# The Administration of Anæsthetics

**Published:** 1919-12-20

**Authors:** 


### THE ADMINISTRATION OF ANÆSTHETICS.

In The Hospital of December 13 we had the opportunity of publishing some excellent and important observations by Dr. Waldo, the City coroner, on the subject of anæsthetic administration. We fully agree with his opinion that the present available data as to the deaths during anæsthesia are so imperfect as to be useless for the purposes of formal investigation, and that in any case no general or local anæsthetic should be administered by anyone but a duly qualified medical man, except in the most exceptional and fully reported circumstances. Also we join the *Times* in congratulating Dr. Waldo on the vigorous way in which he carries on this and other campaigns for more knowledge and better legislation in a number of situations of medical bearing. Undoubtedly the whole question of anæsthetic administration whether by approved or unapproved persons, is one which has, in the past, suffered from considerable neglect and should be—in fact, must be—taken in hand by the Ministry of Health and the Medical Research Committee. It is not only a question of deaths under anæsthesia but of the relative values and best applications of the numerous anæsthetic methods themselves. Several isolated units of investigational work have been lately started and apparently little notice taken of them, owing, perhaps, to the enormous importance of other urgent war considerations which filled much of the surgical horizon, but it is obvious that the neglect must not continue.

In the publication of these various reports and recommendations in the recent Press, we think there are, however, one or two parts which may give the lay reader a somewhat erroneous impression of circumstances occurring in institutional life. It is far from our intention specifically to defend, although one institution was to all intents and purposes specifically accused—and still further from it, to deny or criticise the worth of the coroner's advice to our legislators. We agree the need is obvious, and yet, as we have often pointed out in these columns, it is not always necessary or perhaps wise to invite the layman's criticism of the voluntary hospital or the doctor. Circumstances such as those now considered are not readily understood even after such precise and lucid explanations as those with which Dr. Waldo customarily provides the jury, and still less so when read rapidly and in condensed form from the columns of a daily newspaper. Unfortunately the public is invariably, perhaps inevitably, accustomed to exaggerate the magnitude of such deficiencies reported to it, and, therefore, we think that one or two facts at least which come to mind in reading the coroner's recommendations might also be brought to the public notice.

In the first place, it is not the exception, it is in fact the practically invariable custom for our hospitals to have on their staff, both visiting and resident, well and fully qualified anæsthetists, men of ample experience. Providing that one's guess at the identity of the certain hospital in Southwark be correct, then we can assure the public that this institution has three highly trained visiting anæsthetists, and certainly more than three fully qualified resident ones. Also the question of nurses customarily giving anæsthetics with any frequency in our hospitals may be dismissed as somewhat of a myth except under most exceptionally urgent circumstances, such as midwifery, district work, etc. Coming to the question of student anæsthetists, it must be pointed out that there is a great deal of difference between the public conception of an utterly inexperienced student being told off unaided to anæsthetise a patient and the necessary instructions of the embryo doctor in this part of his art. The custom actually is for the visiting anæsthetist, a man of the greatest experience, daily to initiate two or three students to his methods. These students certainly "hold the chloroform bottle," but both they and the patient are under very careful surveillance meanwhile, and, inasmuch as the "administration of anæsthetics" forms part of the final qualifying examinations for a doctor, we may possibly assume this instruction is necessary, even if the obvious absurdity did not present itself that the said student cannot be a danger to mankind on one day and, his qualification taken, a thoroughly competent anæsthetist the next. We are aware that the true perspective of these facts is perfectly well understood by the profession, but we doubt if the recent lay press comments tend to provide their readers with a similarly proportioned view.

However, it is absurd to suggest either that accidental deaths do not occur under anæsthetics, or that students and nurses never administer chloroform, and we consider that Dr. Waldo is absolutely correct in urging both more precise legislation and more searching investigation work on the whole subject, but at the same time we are not so sure that it is advisable to lead the public to over-criticise either the voluntary or State hospital. Certainly it is unnecessary to direct it upon one institution in particular.

